# Supplementary material for: Quality by Design Based Formulation of Xanthohumol Loaded Solid Lipid Nanoparticles with Improved Bioavailability and Anticancer Effect against PC-3 Cells
Source: Pharmaceutics. 2022 Nov 7;14(11):2403. doi: 10.3390/pharmaceutics14112403 (PMC9699314; doi:10.3390/pharmaceutics14112403)
Supplement: Supplementary file 1 [file pharmaceutics-14-02403-s001.zip › pharmaceutics-1940844-supplementary.pdf]

Supplementary File

Table S1. ANOVA of PS, PDI, ZP, % EE and lack of fit test.

**Summary**								
**Source**	**Sequential *p value***				**Lack of fit *p* value**			
	**R1**	**R2**	**R3**	**R4**	**R1**	**R2**	**R3**	**R4**
**Linear**	< 0.0001	0.0003	0.0555	0.0404	0.7467	0.0473	< 0.0001	< 0.0001
**2FI**	0.6339	0.0091	0.9924	0.324	0.6746	0.1842	< 0.0001	< 0.0001
**Quadratic**	0.2359	0.1821	< 0.0001	< 0.0001	0.8912	0.2433	0.1753	< 0.0001
**Cubic**	0.8912	0.2433	0.1753	< 0.0001				
**Sequential Model Sum of squares (type-1)**								
**Source**	**Sum of squares**				**d*f***			
**Mean Vs total**	401300	0.8856	1276.14	60532.23	1	1	1	1
**Linear Vs mean**	9400.96	0.0132	84.45	3410.74	3	3	3	3
**2FI Vs Linear**	208.82	0.0028	1.01	1132.56	3	3	3	3
**Quadratic Vs 2FI**	510.6	0.0007	109.99	2766.03	3	3	3	3
**Cubic Vs Quadratic**	86.78	0.0004	0.4315	109.3	3	3	3	3
**Residual**	577.3	0.0003	0.208	0.3803	4	4	4	4
**Total**	412100	0.903	1472.22	67951.24	17	17	17	17
**Lack of fit test**								
**Linear**	806.2	0.0039	111.43	4007.89	9	9	9	9
**2FI**	597.38	0.0011	110.42	2875.33	6	6	6	6
**Quadratic**	86.78	0.0004	0.4315	109.3	3	3	3	3
**Cubic**	0	0	0	0	0	0	0	0
**Pure error**	577.3	0.0003	0.208	0.3803	4	4	4	4
**Model summary statistics**								
**Source**	**Standard deviation**				**R-squared**			
**Linear**	10.32	0.0179	2.93	17.56	0.8717	0.7604	0.4307	0.4597
**2FI**	10.84	0.0117	3.33	16.96	0.8911	0.9207	0.4358	0.6124
**Quadratic**	9.74	0.0101	0.023	3.96	0.9384	0.9587	0.9967	0.9852
**Cubic**	12.01	0.0084	0.228	0.3084	0.9465	0.984	0.9989	0.9999

Table S1. (continued): ANOVA of PS, PDI, ZP, % EE and lack of fit test.

**Summary**												
**Source**	**Adjusted R-squared**				**Predicted R-squared**							
	**R1**	**R2**	**R3**	**R4**	**R1**	**R2**	**R3**	**R4**	**R1**	**R2**	**R3**	**R4**
**Linear**	0.8421	0.7051	0.2993	0.3351	0.8007	0.5227	0.0184	0.0341				
**2FI**	0.8257	0.8732	0.0973	0.3798	0.7283	0.6935	-1.0252	-0.3055				
**Quadratic**	0.8593	0.9057	0.9925	0.9662	0.7876	0.5714	0.9631	0.7642				
**Cubic**	0.7859	0.9357	0.9958	0.9998								
**Sequential Model Sum of squares (type-1)**												
**Source**	**Mean square**				**F-value**				**P-value Prob>F**			
**Mean Vs total**	401300	0.8856	1276.14	60532.23								
**Linear Vs mean**	3133.65	0.0044	28.15	1136.91	29.45	13.75	3.28	3.69	< 0.0001	0.0003	0.0555	0.0404
**2FI Vs Linear**	69.61	0.0009	0.3355	377.52	0.5926	6.74	0.0303	1.31	0.6339	0.0091	0.9924	0.324
**Quadratic Vs 2FI**	170.2	0.0002	36.66	922.01	1.79	2.15	401.3	58.84	0.2359	0.1821	< 0.0001	< 0.0001

**Cubic Vs Quadratic**	28.93	0.0001	0.1438	36.43	0.2004	2.1	2.77	383.19	0.8912	0.2433	0.1753	< 0.0001
**Residual**	144.33	0.0001	0.052	0.0951								
**Total**	24238.73	0.0531	86.6	3997.13								
**Lack of fit test**												
**Linear**	89.58	0.0004	12.38	445.32	0.6207	6.2	238.09	4683.65	0.7467	0.0473	< 0.0001	< 0.0001
**2FI**	99.56	0.0002	18.4	479.22	0.6898	2.63	353.91	5040.2	0.6746	0.1842	< 0.0001	< 0.0001
**Quadratic**	28.93	0.0001	0.1438	36.43	0.2004	2.1	2.77	383.19	0.8912	0.2433	0.1753	< 0.0001
**Cubic**												
**Pure error**	144.33	0.0001	0.052	0.0951								
**Model summary statistics**												
**Source**	**Adjusted R-squared**				**Predicted R-squared**				**PRESS**			
**Linear**	0.8421	0.7051	0.2993	0.3351	0.8007	0.5227	0.0184	0.0341	2148.96	0.0083	192.48	7166.04
**2FI**	0.8257	0.8732	0.0973	0.3798	0.7283	0.6935	-1.0252	-0.3055	2929.66	0.0053	397.12	9685.27
**Quadratic**	0.8593	0.9057	0.9925	0.9662	0.7876	0.5714	0.9631	0.7642	2290.47	0.0075	7.23	1749.39
**Cubic**	0.7859	0.9359	0.9958	0.9998								

Method validation

Validation of analytical techniques is important not only for regulatory objectives, but also for their long-term effectiveness and reliability in various research, quality control, process control, product development, clinical and toxicological investigations. This is significant because reliable target variability allows for the detection of atypical behavior of analytical data in routine applications such as quantitative analyte measurement, therefore, increasing data quality reliability and reproducibility. It also proves that a certain analytical approach is dependable, predictable and repeatable. Therefore, the developed analytical method was validated for linearity, accuracy, precision, specificity, robustness, limit of detection (LOD) and limit of quantification (LOQ) as per ICH Q2 (R1) guidelines.

Preparation of quality control samples

Three different concentration levels of quality control samples were prepared i.e. 4.8 µg/ml (LQC, lower quality control), 6µg/ml (MQC, middle quality control) and 7.2 µg/mL (HQC, high quality control). The samples were stored at 4°C for further analysis. The prepared samples were passed through 0.22 µm syringe filter before proceeding to chromatography.

Linearity

The linearity of analytical method was evaluated with the prepared samples of concentration 2-10µg/mL from the stock solution (1000µg/mL) with n=6. The linearity curve was plotted by taking peak area (mAU) on Y axis and concentration of XH (µg/mL) on X axis. Slope, regression equation and regression coefficient (r^2) were calculated for linearity curve by using MS-Excel software [1].

LOD and LOQ

The determination of LOD and LOQ for the method was performed at 3:1 and 10:1 signal to noise ratio by using standard deviation (σ) and slope of the standard curve [2]. LOD and LOQ were calculated using the formula given in Eq (1):

$$LOD = 3.3 * \sigma / S \text{ and } LOQ = 10 * \sigma / S \qquad \text{Eq (1).}$$

Where, σ is standard deviation and S is slope of the standard curve.

Accuracy

Accuracy of the method was studied based on the absolute recovery of XH using its LQC, MQC and HQC concentrations that indicate recovery at 80%, 100% and 120% respectively. The experiment was carried out in hexaplicate and the mean data, standard deviation (SD or σ), % relative standard deviation (% RSD) and % absolute recovery were calculated for each sample to confirm the accuracy data within specified limits [3]. The % absolute recovery was calculated using the formula given in Eq (2).

$$\% \text{ Absolute recovery} = \frac{Actual\ concentration\ recovered}{Theoritical\ concentration} X\ 100 \qquad \text{Eq (2)}$$

Precision

The consistency of results among several distinct aliquots of the identical concentration on the same day and other days of the analysis is explained by the precision of the developed analytical method. Intraday (repeatability), inter-day (reproducibility) and inter-analyst precision of the developed method was analyzed by injecting (six times) three diverse concentrations (LQC, MQC and HQC) of XH on initial day and three successive days under similar experimental conditions. The inter-analyst precision study was carried out by injecting hexaplicate samples of LQC, MQC and HQC by three different analysts on the same day. Mean data, SD, % RSD, recovery was computed [2].

Robustness

Robustness is a measure of analytical technique consistency; it is method's ability to stay unchanged by modest purposeful alterations in parameters of the method. Robustness of the analytical method was analyzed by applying variations in flow rate (1.0, 0.8, 0.6 mL/min) and wave length (365, 370 and 375 nm). MQC (6µg/ml) solution of XH was used for the experiment and its mean area, mean retention time and % RSD were calculated for analyzing the changes that took place in the chromatogram [4–7].

System suitability

System suitability testing is a method of ensuring a chromatographic system's appropriateness for a specific analysis by verifying its resolution, column efficiency, and repeatability. Suitability test is predicted on the premise that the equipment, analytical procedures, electronics and samples to be analyzed are all part of a larger system that can be evaluated. This test was performed by injecting six injections of 10 µg/mL working solutions. %RSD, peak area, retention time, theoretical plates, tailing factor, peak purity index and height equivalent to theoretical plate (HETP) were determined and compared with their official limits [6,7].

Results of Method validation

Linearity

The results of calibration curve **(Figure S1)** deciphered that XH has obeyed good linearity with the working standard solutions in the range of 2-10µg/mL. The calibration curve was found to be linear, with a decent regression coefficient (r^2) of 0.9996.

A)

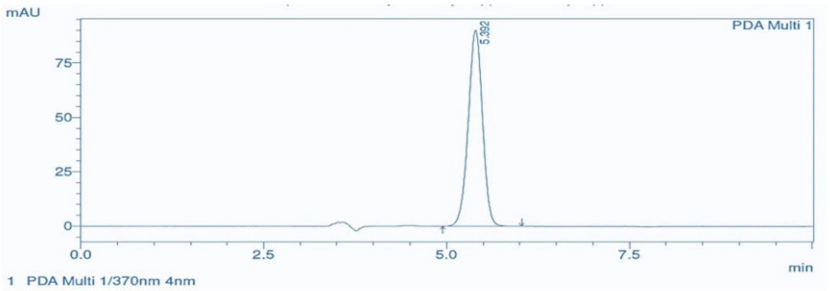

B)

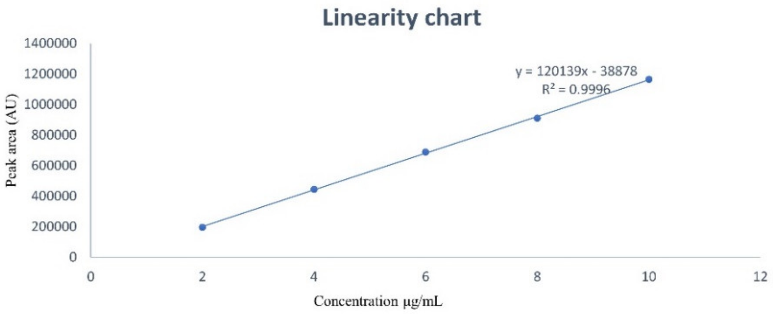

Figure S1. Depicting the chromatograms of A) Xanthohumol and B) Linearity chart of XH.

LOD and LOQ

The method has extremely low LOD and LOQ values of 0.425µg/mL and 1.289 µg/mL respectively, suggesting that the proposed method for XH estimation has a high sensitivity.

Accuracy (Recovery method)

Accuracy of the method was performed by recovery method, the recovery for LQC, MQC and HQC was appreciable and % recovery was found to be in between 101.1% to 109.7%. The % RSD values were less than 2. Therefore, the results were found to be within limits, which indicated that the developed method for estimation of XH has accuracy of high degree. The results of accuracy study are shown in **Table S2.**

Table S2. Representing accuracy data.

**Levels**	**Concentration of standard (µg/ml)**	**Concentration of sample (µg/ml)**	**Mean area ± SD (n=6)**	**%RSD**	**% Recovery Mean area ± SD**	**%RSD**
**LQC**	4.8	6	598171.3±1850.811	0.309	109.7±1.084	0.988
**MQC**	6	6	690278.2±5265.435	0.762	101.1±0.769	0.761
**HQC**	7.2	6	843344.6±7804.514	0.925	101.95±0.900	0.883

Precision

The precision of method was determined by using three quality control standards (LQC, MQC and HQC) and the obtained results of the study were shown in the **Table S3**. The % RSD of inter-day (0.64%-1.10%), inter-analyst (0.594%-0.889%) and intra-day (0.738%-0.994%) precision was less than 2% which indicated that the results were within the acceptable limits and the developed method was precise.

Table S3. Representing precision data.

Levels	Concentration (µg/ml)	Parameters: Interday precision (Repeatability) (Mean area ± SD) (n=6)	%RSD	Interanalyst (mean area ± SD) (n=6): Analyst 1, Analyst 2, Analyst 3	%RSD	Intraday precision (mean area ± SD) (n=6): Day 1, Day 2, Day 3	%RSD
**LQC**	4.8	596769.7±4592.902	0.76	596278.4±3545.17	0.594	590802.8±5873.300	0.994
**MQC**	6	691518.4±4491.639	0.64	690742.9±4967.83	0.719	690659.7±5102.69	0.738
**HQC**	7.2	840356.3±9244.591	1.10	843885.7±7510.5	0.889	870089.2±6965.64	0.800

Robustness

The robustness of the method was performed by changing the wavelength (365, 370 and 375 nm) and flow rate (0.6, 0.8, 1.0 mL/min). The results of % RSD were found to be less than 2%, which indicated that the method was robust. The results of robustness are shown in **Table S4.**

Table S4. Representing Robustness data of the method.

Variable	Value (ml/min)	Concentration (µg/ml)	(Mean area ± SD) (n=6)	%RSD	(Mean R_t ± SD) (n=6)	%RSD
**Flow rate (ml/min)**	0.6	6	886757.4 ±11624.28	1.310	6.9±0.027	0.403
	0.8	6	648420.8±5814.9	0.896	5.1±0.039	0.756
	1	6	607624.1±2930.1	0.482	4.1±0.050	1.212
**Wave-length**	365	6	613765.6±2492.7	0.4	5.1±0.04	0.931
	370	6	690351.9±5983.351	0.931	5.1±0.03	0.588
	375	6	706587.3±6579.4	0.931	5.1±0.051	1.0

Parameters of system suitability

After six injections of 10µg/mL, the system suitability findings revealed that there was no significant change in the critical attributes i.e., peak area, retention time, theoretical plates, peak purity index and peak tailing factor of XH. System suitability parameters are shown in **Table S5**.

Table S5. Depicting system suitability parameters.

Parameter	Value	Limit
**Tailing factor**	0.991	<2
**Theoretical plate**	4446.667	>2000
**HETP**	31.7	Depends on theoretical plate
**Peak purity index**	0.999	>0.5

System specificity

The system specificity was assessed by injecting the blank samples of all the excipients used in the preparation of SLN's. No significant peak was observed at the retention time of pure XH. Hence, the developed method was found to be specific for XH. The chromatograms of all the excipients were shown in **Figure S2**.

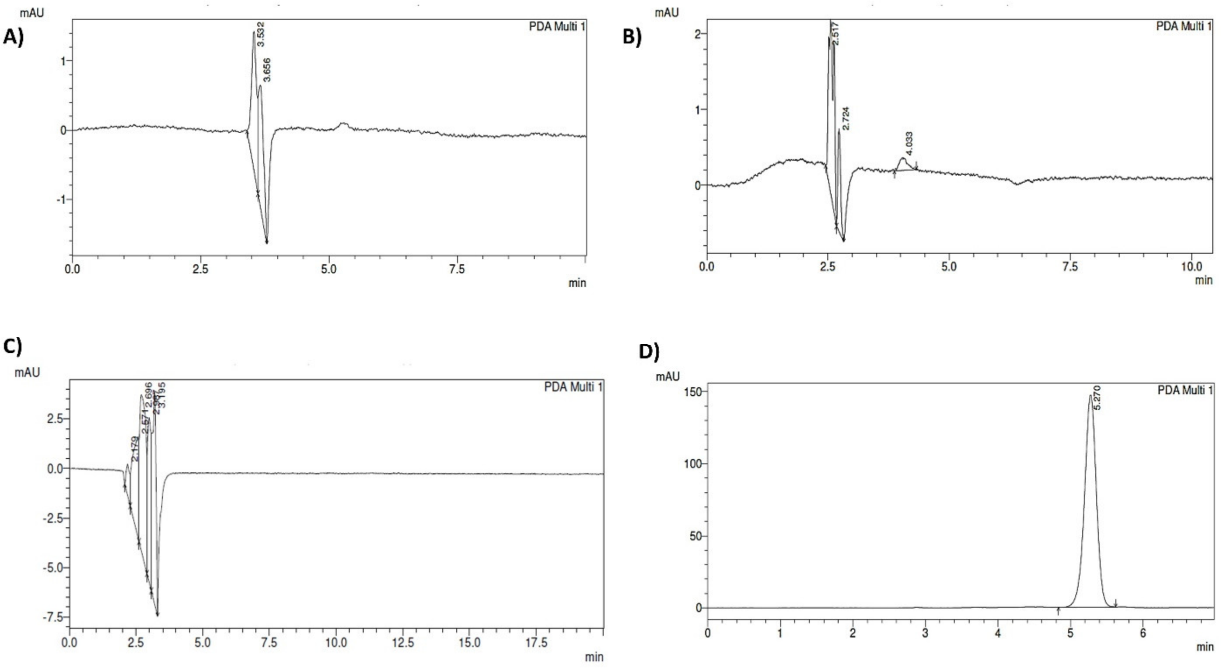

Figure S2. Depicting the chromatograms of A) blank compritol E ATO B) blank pluronic F 68 C) blank lipoid S E80 D) XH loaded SLN.

References

1. Pecchio, M.; Salman, H.; Irache, J.M.; Renedo, M.J.; Dios-Viéitez, M.C. Development and Validation of a HPLC Method for the Determination of Cyclosporine a in New Bioadhesive Nanoparticles for Oral Administration. *Indian J. Pharm. Sci.* **2014**, *76*, 132.
2. Ameeduzzafar; El-Bagory, I.; Alruwaili, N.K.; Imam, S.S.; Alomar, F.A.; Elkomy, M.H.; Ahmad, N.; Elmowafy, M. Quality by Design (QbD) Based Development and Validation of Bioanalytical RP-HPLC Method for Dapagliflozin: Forced Degradation and Preclinical Pharmacokinetic Study. *J. Liq. Chromatogr. Relat. Technol.* **2020**, *43*, 53–65.
3. Singh, G.; Pai, R.S.; Pandit, V. Development and Validation of a HPLC Method for the Determination of Trans-Resveratrol in Spiked Human Plasma. *J. Adv. Pharm. Technol. Res.* **2012**, *3*, 130.
4. Dejaegher, B.; Heyden, Y. Vander Ruggedness and Robustness Testing. *J. Chromatogr. A* **2007**, *1158*, 138–157, doi:10.1016/j.chroma.2007.02.086.
5. Kumar, R.; Kumar, R.; Khursheed, R.; Awasthi, A.; Khurana, N.; Singh, S.K.; Khurana, S.; Sharma, N.; Gunjal, P.; Kaur, J. Development and Validation of RP-HPLC Method for Estimation of Fisetin in Rat Plasma. *South African J. Bot.* **2020**.
6. Kumar, R.; Kumar, R.; Khursheed, R.; Awasthi, A.; Ramanunny, A.K.; Kaur, J.; Khurana, N.; Singh, S.K.; Khurana, S.; Pandey, N.K. Validated Reverse Phase-High-Performance Liquid Chromatography Method for Estimation of Fisetin in Self-Nanoemulsifying Drug Delivery System. *Assay Drug Dev. Technol.* **2020**, *18*, 274–281.
7. Kapoor, B.; Gupta, R.; Gulati, M.; Singh, S.K.; Khatik, G.L.; Chawla, M.; Nagappan, K.V.; Khursheed, R.; Kumar, R. High-Performance Liquid Chromatography and Liquid Chromatography/Mass Spectrometry Studies on Stress Degradation Behavior of Sulfapyridine and Development of a Validated, Specific, Stability-Indicating HPLC Assay Method. *Assay Drug Dev. Technol.* **2020**, *18*, 119–133.
